# The Bureau of Animal Industry

**Published:** 1884-10

**Authors:** 


### THE BUREAU OF ANIMAL INDUSTRY.

WASHINGTON, D. C., July 26, 1884.—All matters relating to the cattle quarantine are now in process of transfer from the Treasury Department to the newly created Bureau of Animal Industry in the Agricultural Department.

The above clipping from a Boston daily contains news which may yet be of the utmost importance to the future of Veterinary Medicine in the United States. One of the most important facts which it conveys to us is that the Treasury Cattle Commission is soon to be relegated to that obscurity which it well deserves.

We are certainly more happy at these changes than any member of the profession; happy for the sake of our future, though we cannot see that it will in any way serve us personally.

The profession, again, may congratulate itself that for the first time in its history in this country it is to be represented at Washington by a man in whom we may all have confidence, and to whom we can all point with pride. The work which we have thus far seen of Dr. Salmon indicates that he is a man of true scientific spirit, which can only emanate from an enthusiastic brain; and the evidences of history have all been such as to warrant us in assuming that devotion to research, and enthusiasm for the profession which he so well honors, will be the characteristics which we shall see portrayed in his career.

The mutual sympathy which, like a mystic wand, binds men of scientific ambitions together, necessitates our having the strongest confidence in Dr. Salmon, and to feel sure that it will not be misplaced. He has now, however, to demonstrate that he possesses other qualities than those suited to the laboratory alone. Being the technical head of the Bureau, he must realize that his organizative talent is to be tested, for the credit or discredit of the work done will fall on its head, rather than upon the detail workers. Real enthusiasm for the advancement of the profession is rather a rare article in this land, where Dollar is King; yet it is only the possessors of it that can do the best work for the State as public officers. The work on the Diseases of our Domestic Animals, which has emanated from the Department of Agriculture, has been exceptionally good; and while we have never failed to show where it was incomplete, or where it appeared to be erroneous, and occasionally have felt it our duty to take the Commissioner to task, still we have ever felt that Dr. Loring was doing a good work, better perhaps than would be even hoped for under the restrictive influences of American politics. We hope much for the future if the Commissioner can only retain his office under the next Administration, and can but say that it will be almost impossible to make any change in this office which will not materially injure our Stock Raising interests, and retard our professional advancement.

As we said in our last issue, the Bureau of Animal Industry can be made to be the nucleus of a system of Veterinary Police and Animal and Public Hygiene in the country. One of its outcomes should be the formation of similar bureaus in each State, having work of a like nature to perform. The members of the profession in each State must consider it a personal duty to advocate this work.

Such an organization over the land should be the means of stimulating each student to educate himself as thoroughly as possible; for such positions should be impossible of attainment through *influence* in any form, but should be won by public competition, the best man winning, except in cases where individual men have shown their fitness by exceptionally valuable work; and even here we advocate competitive examination. We shall never be satisfied until we see a Veterinarian-in-Chief at Washington, a similar one in every State, and County and District Veterinarians, with Market Inspector and such other local officials as the public necessities may demand.

We have endeavored faithfully, and almost single-handed, to lay the foundation of such a Veterinary Police System for the country, and hence it is that this Bureau of Animal Industry appears as the rising sun of hope, illuminating a hitherto dark and hopeless horizon. B.

